# Diseases of the Spinal Cord

**Published:** 1886-07

**Authors:** 


DISEASES OF THE SPINAL CHORD. *By* BYROM BRAMWELL, M. D., F. R. C. P. (*Edin.*): *Lecturer on the Principles and Practices of Medicine, and on Medical Diagnosis, in the Extra Academical School of Medicine, Edinburgh; Pathologist to the Edinburgh Royal Infirmary; Additional Examiner in Clinical Medicine in the University of Edinburgh; late Physician and Pathologist to the Newcastle-on-Tyne Infirmary; formerly Medical Officer to the Tynemouth Union Workhouse Hospital, the Prudhoe Memorial Convalescent Home, the Tyne Floating Hospital, etc., etc. Fifty-three colored plates, and one hundred and two fine wood engravings. Second edition. Octavo, pp. xiv, 298. New York:* WILLIAM WOOD AND COMPANY. *Chicago:* W. T. KEENER.

The appearance of a second edition of this work within so short a time, and its translation into the German, French and Russian languages, is presumptive evidence of its value. The German edition is the most popular text-book on the subject in use in that country.

This last edition is somewhat enlarged, and contains thirty-two additional engravings and plates. These serve to further illustrate the clear and accurate text. The addition of a chapter on concussion of the spine, and the clinical examination of "railway cases" is timely, in view of the forensic importance of these conditions.

Among the original illustrations we are pleased to note that the old favorite, "an acute bed-sore (after Charcot)" is not forgotten. We trust that beginners in this field will not infer that acute bed-sores are rare, from the fact that there has never been but one illustration for them.

